# Early-Life Exposure to the Cooking Oil Fume Component *trans*,*trans*-2,4-Decadienal Impairs Ocular Development and Angiogenesis in Zebrafish (*Danio rerio*) Larvae

**DOI:** 10.3390/toxics14050388

**Published:** 2026-04-30

**Authors:** Xiaoli Wu, Xinyue Zhang, Zengliang Ruan

**Affiliations:** 1 Key Laboratory of Environmental Medicine and Engineering of Ministry of Education, School of Public Health, Southeast University, Nanjing 210096, China; 220244229@seu.edu.cn (X.W.); xyz@seu.edu.cn (X.Z.); 2 Department of Epidemiology and Health Statistics, School of Public Health, Southeast University, Nanjing 210096, China; 3 Department of Medical Epidemiology and Biostatistics, Karolinska Institutet, 171 77 Stockholm, Sweden

**Keywords:** *trans*,*trans*-2,4-decadienal, environmental pollution, histopathology, vascularization, laboratory fish, cytotoxicity

## Abstract

*Trans*,*trans*-2,4-decadienal (*tt*-DDE), the primary aldehyde component found in cooking oil fumes, is a prevalent environmental pollutant. However, its potential adverse effects on ocular development remain largely unexplored. This study evaluated its toxicity on ocular development and angiogenesis in zebrafish larvae, as well as on human retinal vascular endothelial cells (HRECs). Zebrafish (*Danio rerio*) larvae at 48 h post-fertilization were microinjected intraocularly with various doses of *tt*-DDE (65.87–521.3 mM) for 24 h. We observed dose-dependent impairments in ocular development following *tt*-DDE exposure. It significantly reduced eye size and inhibited the intraocular vascular area at concentrations of 128.9 mM and above. Histopathological analysis revealed retinal structural disorganization, eye shrinkage, and a clear dose-dependent increase in acridine orange (AO) fluorescence intensity. Apoptosis assays confirmed a significant escalation in ocular cell death at higher exposure doses. Additionally, our results demonstrated that *tt*-DDE (5–100 μM) significantly reduced the viability of HRECs in vitro. These findings suggest that early-life exposure to *tt*-DDE impairs ocular development in zebrafish by inducing histopathological damage, inhibiting angiogenesis, and promoting apoptosis, and also exerts direct cytotoxicity to human retinal cells. This study underscores the potential risk of *tt*-DDE exposure as an environmental factor contributing to ocular developmental toxicity.

## 1. Introduction

Cooking oil fumes (COF) are a complex chemical mixture created through a series of thermodynamic reactions between fats, lipids, and organic matter, which typically contains a diverse range of toxic chemical components such as ketones, aldehydes, alkanes, alkenes, fatty acids, alcohols, esters, aromatic and heterocyclic compounds [1,2,3]. Among these components, aldehydes emerge as the major constituent, with *trans*,*trans*-2,4-decadienal (*tt*-DDE), a distinct variant of *α*,*β*-unsaturated aldehyde, being one of the most abundant subtypes within COF [4]. *tt*-DDE is an important byproduct resulting from the peroxidation of linoleic acid and polyunsaturated lipids during cooking and is commonly used as a food additive and flavoring agent, resulting in potentially widespread human exposure [5,6].

Previous research has shown an association between *tt*-DDE and various health outcomes, particularly the occurrence and development of cardiovascular, respiratory, and alimentary system dysfunctions [7,8,9]. For example, *tt*-DDE may increase the risk of hypertension and the enlargement of atherosclerotic lesions by triggering oxidative or nitrative stress-related damage and cell apoptosis in the arteries of rats [10,11]. Recent evidence has demonstrated that *tt*-DDE exerts direct cytotoxic effects on human umbilical vascular endothelial cells (HUVECs) through oxidative stress-mediated disruption of mitochondrial and autophagic pathways [5]. In addition, the epithelial hyperplasia and granulomatous nodules at the bronchiol-alveolar junction, which are known risk factors associated with lung carcinogenesis, were significantly increased in *tt*-DDE-exposed mice in a dose–response manner [12]. Consistent with these findings, a study reported that *tt*-DDE induces DNA damage in human bronchial epithelial cells (BEAS-2B), further supporting its genotoxicity in human respiratory cells [9]. Furthermore, *tt*-DDE has been reported to have a potent inhibitory effect on gastric emptying by directly stimulating the serotonin-producing enteroendocrine cells [13]. More recently, an in vitro study has shown that it can significantly induce cytotoxic responses and mitochondrial damage in human corneal epithelial (HCE) cells, highlighting its potential risk of ocular damage [14]. Although *tt*-DDE, as a major component in cooking oil fumes, can come into direct contact with the eyes, its ocular toxic effects remain insufficiently investigated.

Zebrafish (*Danio rerio*) serve as a sensitive, cost-effective, and versatile model for biomedical research due to their extensive physiological and genetic similarities to humans [15,16,17]. For example, a diabetic retinopathy model can be easily developed through short-term exposure to high-glucose conditions using transgenic zebrafish larvae, which is useful for screening and drug discovery [18]. In addition, zebrafish is particularly useful for exploring the in vivo toxicity of environmental hazardous materials during ocular development, aligning with the principles of replacement, reduction, and refinement (the 3Rs) in animal research [19].

Given the high susceptibility of ocular tissues to environmental toxicants and the potential of *tt*-DDE to act directly on the ocular surface [14,20], the objective of this study is to provide initial evidence for the intrinsic retinotoxic potential of *tt*-DDE by evaluating its effects on ocular development and angiogenesis in zebrafish larvae in vivo. To achieve this, we employed intraocular microinjection, a mature approach in ocular toxicology that enables precise dose control while circumventing systemic metabolic interference. This method has been well validated in ocular toxicology for assessing retinal toxicity, oxidative stress, and angiogenesis [21,22,23]. For example, evidence from systematic comparisons of immersion, intraperitoneal injection, and intravitreal injection indicates that the latter is the preferred method for establishing acute retinal damage models in zebrafish [23]. In addition, we further explored the underlying mechanisms involved in the biological effects of *tt*-DDE in human retinal vascular endothelial cells (HRECs).

## 2. Materials and Methods

### 2.1. Chemicals

*Trans*,*trans*-2,4-decadienal (*tt*-DDE, CAS#: 25152-84-5) was purchased from Aladdin Biochemical Technology (Shanghai, China). Dimethyl sulfoxide (DMSO) was obtained from Sigma (St. Louis, MO, USA). Acridine orange (AO) and xylene were bought from Macklin Biochemical (Shanghai, China). *tt*-DDE was dissolved in DMSO as the vehicle and diluted as needed. Phosphate-buffered saline (PBS) was purchased from Procell Life Science & Technology Co., Ltd. (Wuhan, China). Sodium chloride injection was purchased from Hunan Kelun Pharmaceutical (Yueyang, China). Methyl cellulose was bought from Aladdin Biochemical Technology. Absolute alcohol and ammonium hydroxide were bought from Sinopharm Chemical (Shanghai, China). Paraformaldehyde, 4% and neutral balsam were purchased from Solarbio Science & Technology (Beijing, China). Eosin and hematoxylin staining solutions were bought from Shanghai Yihe Biotechnology (Shanghai, China). Dilute hydrochloric acid was bought from Shenzhen Bolinda Technology (Shenzhen, China). High-efficiency sliced paraffin wax was bought from Huayong Paraffin (Shanghai, China).

### 2.2. Zebrafish Husbandry and Exposure to tt-DDE

Wild-type (WT) zebrafish (AB strain) and transgenic (Tg) fluorescent zebrafish (fli1: enhanced green fluorescent protein, EGFP) were obtained from Hunter Biotechnology (Hangzhou, China) and were raised in fish culture water at 28 °C (200 mg Instant Sea Salt per 1 L of reverse osmosis water, conductivity at 450~550 μS/cm; pH was 6.5~8.5; 50~100 mg/L for CaCO_3_), and reproduction was carried out in natural pairs. At 48 h post-fertilization (hpf), morphologically normally developing zebrafish are randomly selected and transplanted into 6-well plates (30 larvae per well). *tt*-DDE was injected into zebrafish eyes using a microinjector (IM300, Narishige, Tokyo, Japan) at 28 °C and exposed for 24 h, while setting up a normal control (intraocular injection of sterile saline) and solvent control group (intraocular injection of DMSO). Prior to injection, larvae were anesthetized with 0.02% (*w*/*v*) tricaine methanesulfonate (MS-222, Sigma, St. Louis, MO, USA), and each injection was completed within 30 s. After injection, larvae were immediately transferred to fresh system water for recovery. This intraocular route enabled precise dose delivery to the target tissue, minimizing systemic confounders and allowing accurate assessment of local ocular toxicity. According to the preliminary mortality data (Table S1 and Figure S1), the maximum non-lethal dose (MNLD) and 10% lethal dose (LD_10_) were 0.5213 M and 0.8191 M under the protocols we used. The final exposure doses were set at 65.87, 128.9, 260.6, and 521.3 mM (i.e., 1/8 MNLD, 1/4 MNLD, 1/2 MNLD, and MNLD), each delivered in a volume of 2 nL/tail. All experiments were performed at Hunter Biotechnology with three replicates, and treatments were conducted in compliance with standard ethical guidelines.

### 2.3. Morphological Development of the Eye

After exposure to *tt*-DDE, we randomly selected ten zebrafish from each group and photographed them using a dissecting microscope (SZX7, OLYMPUS, Tokyo, Japan) under brightfield illumination at 80× magnification. For each larva, the lateral image of the right eye was used for analysis. The region of interest (ROI) was defined as the entire eye area, determined by tracing the eye boundary on the lateral image. Imaging conditions were kept identical for all samples. Eye area measurements were performed using the advanced image processing software NIS-Elements D 3.20.

### 2.4. Evaluation of Ocular Vascular Area

Ten transgenic *Tg (fli1:EGFP)* zebrafish larvae were randomly selected after *tt*-DDE exposure and subjected to fluorescence microscopy for quantification of intraocular vascular area. For imaging, larvae were positioned transversely on glass slides, and their ocular vasculature was visualized using a fluorescence microscope (AZ100, Nikon, Tokyo, Japan) under green fluorescence with an exposure time of 500 ms, gain of 6.4×, and magnification of 160×. Image analysis was performed using NIS-Elements D 3.20 advanced image processing software to measure the total area of fluorescently labeled blood vessels.

### 2.5. Histopathological Analysis

Zebrafish from each treatment group were fixed in 4% paraformaldehyde at 4 °C for 24 h. After fixation, the specimens were washed twice with PBS and then dehydrated through a graded ethanol series (70%, 80%, 95%, 100% absolute ethanol and a 1:1 mixture of xylene and ethanol) for a total of 100 min. The dehydrated specimens were manually embedded in paraffin wax and sectioned at 3–5 μm thickness using a microtome (KD2258, Jinhua Kedi Medical Equipment Co., Ltd., Jinhua, China). Sections were stained with Hematoxylin and Eosin (H&E) following standard protocols. The ROI was defined as the entire area of the eye, excluding surrounding non-ocular tissues. For each treatment group, three randomly selected zebrafish were examined under a biological microscope (CX31, Olympus, Tokyo, Japan) at 10× eyepiece magnification, and the pathological changes in ocular tissues were assessed qualitatively.

### 2.6. Determination of Ocular Cell Apoptosis

To quantify apoptosis levels in the zebrafish eyes, *tt*-DDE-exposed zebrafish larvae were rinsed twice with PBS (pH 7.4) and subsequently transferred to another PBS solution containing AO for staining. The larvae were then maintained in the dark at standard room temperature for 15 min, followed by three sequential washes in the same PBS buffer. Ten WT zebrafish larvae were randomly selected and imaged using a fluorescence microscope (Nikon AZ100, Tokyo, Japan) with a navy excitation filter. AO staining was used as a semi-quantitative indicator of increased membrane permeability and nuclear condensation, which are associated with apoptotic or necrotic processes [24]. Fluorescence intensity was measured using NIS-Elements D 3.20 software, and the mean intensity per eye region was compared across groups. Imaging conditions were kept identical for all samples.

### 2.7. Cell Culture and Cytotoxicity Assay

The human retinal vascular endothelial cells (HRECs, Procell Life Science & Technology Co., Ltd., Wuhan, China) were cultured in DMEM (Cytiva, Marlborough, MA, USA) containing 10% (*v*/*v*) fetal bovine serum (FBS, Cytiva, Marlborough, MA, USA), 100 µg/mL streptomycin, and 100 U/mL penicillin (Cytiva, Marlborough, MA, USA) in an incubator with 5% CO_2_ at 37 °C. The culture medium was renewed twice a week, and cells were subcultured every 72 h via trypsinization. Before treatment, they were plated at a density of 8 × 10^4^ cells/well in 96-well plates (Corning, NY, USA) and maintained overnight to ensure cell attachment. After incubation, the culture medium was renewed with different levels of *tt*-DDE (5, 10, 20, 50, 100 μM) for 24 h. DMSO with a final concentration of 0.05% was used as the solvent control, and a blank control (culture medium without cells) was included for background subtraction. Cytotoxicity of *tt*-DDE was determined with the CCK-8 assay (Abcam Inc., Cambridge, UK). Following exposure, the medium was replaced, and CCK-8 reagent was added at a 1:10 dilution (*v*/*v*). After 2 h at 37 °C, absorbance at 450 nm was recorded with the full-wavelength MicroplateReader Eyes (YoMim, Hangzhou, China). Cell viability (%) was calculated based on absorbance values at 450 nm (OD_450_) using the formula: (OD_450_ of treated group − OD_450_ of blank control)/(OD_450_ of solvent control group − OD_450_ of blank control) × 100%. All experiments were performed with a minimum of three independent biological replicates.

### 2.8. Statistical Analysis

The quantitative variables are shown as mean ± standard error of the mean (SEM) for parametric data or as median ± interquartile range (IQR) for non-parametric data. Data were analyzed using SPSS (IBM Statistics SPSS 26.0) and GraphPad Prism 10.1.2. After verifying the assumptions of normality (Shapiro–Wilk test) and homogeneity of variances (Levene’s test), group comparisons were performed using one-way analysis of variance (ANOVA) followed by Dunnett’s post hoc test for multiple comparisons against the control group. When the parametric assumptions were not met, the Kruskal–Wallis non-parametric test and Dunn’s post hoc test were used for group comparisons. The differences were considered statistically significant at *p* < 0.05.

## 3. Results

### 3.1. The Effect of tt-DDE on Zebrafish Eye Area

**Figure 1:**
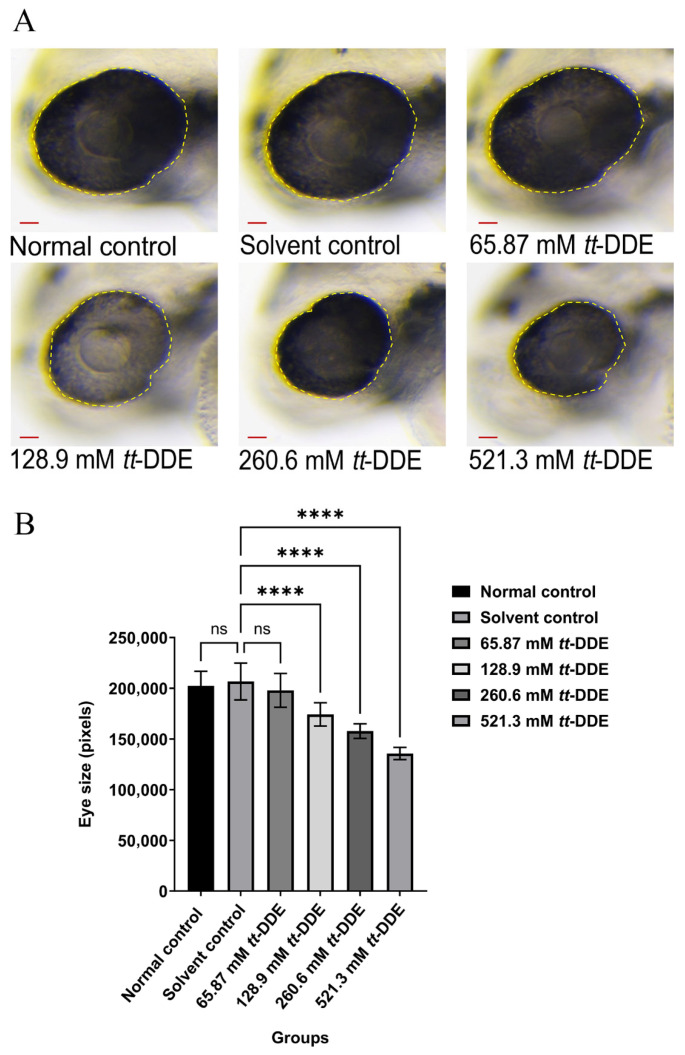
The effect of *tt*-DDE on the zebrafish eye area. Wild-type AB zebrafish were ocularly injected with 65.87 mM, 128.9 mM, 260.6 mM, and 521.3 mM of *tt*-DDE; the normal control and the solvent control group were injected with saline and DMSO, respectively. (**A**) Representative microscopic images of zebrafish eyes (magnification 80×). The eye region of each larva is outlined by a yellow dashed line, scale bar = 100 μm. (**B**) Quantification of eye area (in pixels) for each treatment group. Data are presented as mean ± SEM, *n* = 10 larvae per group. Statistical comparisons between each *tt*-DDE treated group and the solvent control were performed using one-way ANOVA followed by Dunnett’s post hoc test. **** *p* < 0.0001 compared with the solvent control group; ns, not significant (*p* > 0.05).

The results presented in Figure 1 illustrate the impact of *tt*-DDE on zebrafish eye area, with microscopic images showing the eye regions under various treatments, including normal control, solvent control, and different concentrations of *tt*-DDE. The results demonstrate that exposure to *tt*-DDE induced a severe and dose-dependent reduction in eye size in zebrafish, with almost all treated groups (128.9, 260.6, and 521.3 mM) showing a statistically significant decrease in eye area (*n* = 10, *p* < 0.001) compared to the solvent control, which itself showed no effect relative to the normal control.

### 3.2. Inhibition of Ocular Vasculature by tt-DDE in Zebrafish

**Figure 2:**
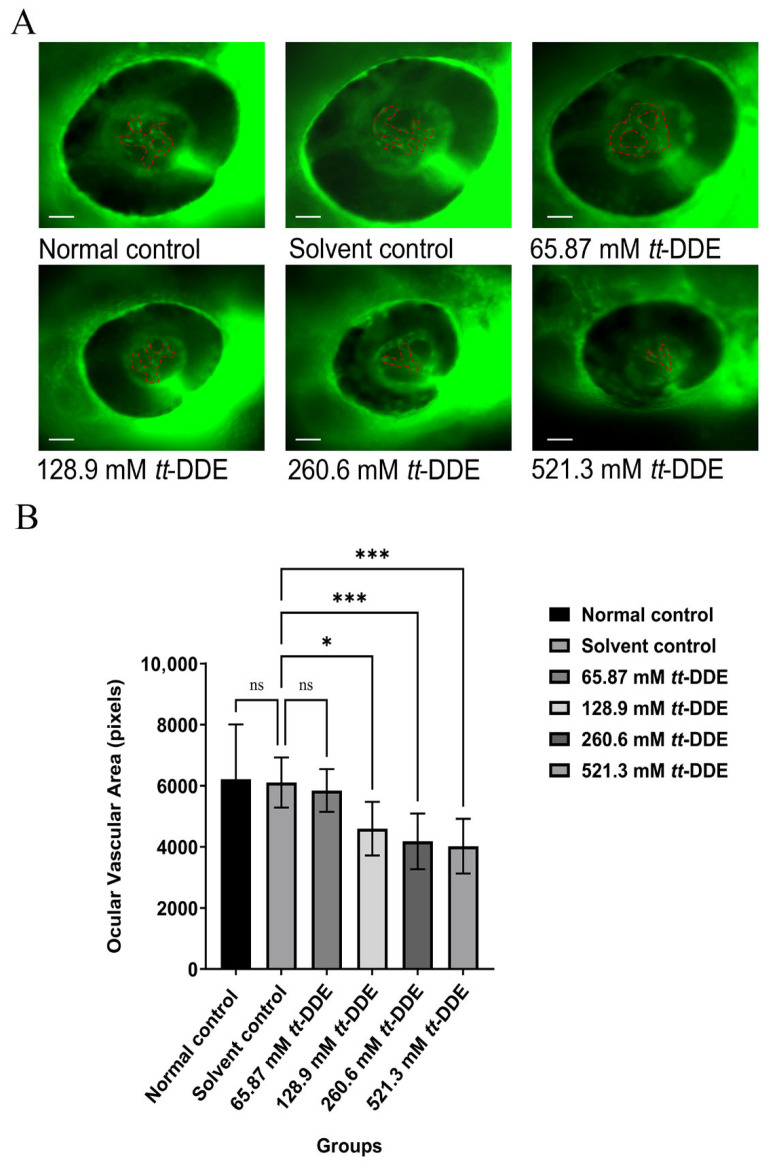
The impact of *tt*-DDE on the area of blood vessels in the zebrafish eye. *fli1:EGFP* zebrafish were ocularly injected with 65.87 mM, 128.9 mM, 260.6 mM, and 521.3 mM of *tt*-DDE; the normal control and the solvent control group were injected with saline and DMSO, respectively. (**A**) Representative fluorescence images of zebrafish eyes showing ocular vasculature (magnification 160×). The intraocular blood vessels are outlined by red dashed lines, scale bar = 100 μm. (**B**) Quantitative analysis of intraocular vascular area (in pixels) within the eye region across treatment groups. Data are presented as mean ± SEM, *n* = 10 larvae per group. Statistical comparisons between each *tt*-DDE treated group and the solvent control were performed using one-way ANOVA followed by Dunnett’s post hoc test. * *p* < 0.05, *** *p* < 0.001 compared with the solvent control group; ns, not significant (*p* > 0.05).

Exposure to *tt*-DDE resulted in a pronounced dose-dependent inhibition of ocular vascular development in zebrafish, as illustrated in Figure 2A and further supported by quantitative analysis in Figure 2B. Treatment with the compound at 128.9, 260.6, and 521.3 mM led to a significant decrease in eye vasculature area compared to the solvent control group, which was indistinguishable from the normal control, indicating that the observed anti-angiogenic effects were directly attributable to the compound.

### 3.3. Ocular Histopathological Alterations

**Figure 3:**
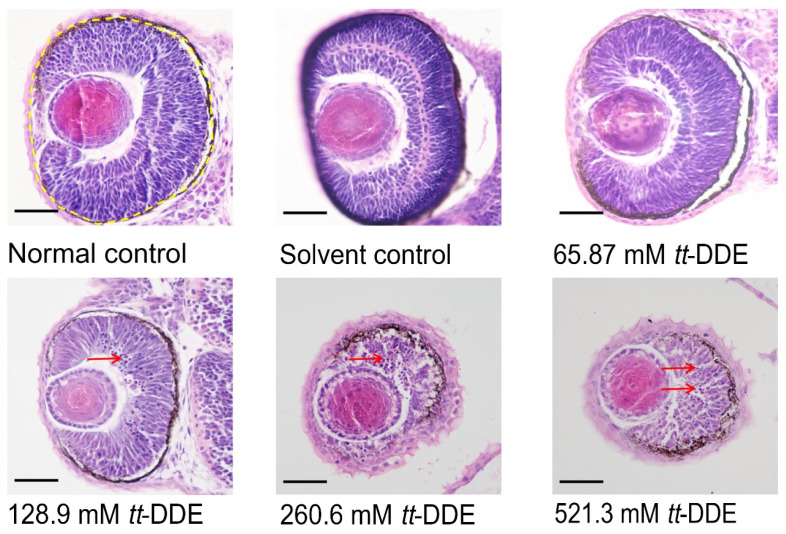
Representative H&E stained sections of the zebrafish eye (magnification 400×). Wild-type AB zebrafish were ocularly injected with 65.87 mM, 128.9 mM, 260.6 mM, and 521.3 mM of *tt*-DDE; the normal control and the solvent control group were injected with saline and DMSO, respectively. The entire eye region is outlined by a yellow dashed line; red arrows indicate apoptotic cells, characterized by nuclear pyknosis, chromatin condensation, and margination. Scale bar = 100 μm.

Based on the H&E staining of zebrafish eye sections (Figure 3), exposure to *tt*-DDE induced significant histopathological alterations and a clear dose-dependent increase in apoptotic cells within the eye tissue. The normal and solvent control groups display typical ocular morphology with well-organized retinal layers and no apparent cellular abnormalities. In contrast, zebrafish eyes exposed to *tt*-DDE exhibit noticeable histopathological changes in the eye morphology, including eye shrinkage, unclear retinal structure, and the presence of apoptotic cells in the retina. These changes are evident across all tested concentrations (128.9, 260.6, and 521.3 mM), with increasing severity correlating to higher doses. The yellow outlines demarcate the retinal regions, highlighting the structural alterations and cellular damage induced by *tt*-DDE. The findings suggest that *tt*-DDE induces dose-dependent retinal toxicity in zebrafish, characterized by increased cellular apoptosis.

### 3.4. Apoptosis in the Zebrafish Eye

**Figure 4:**
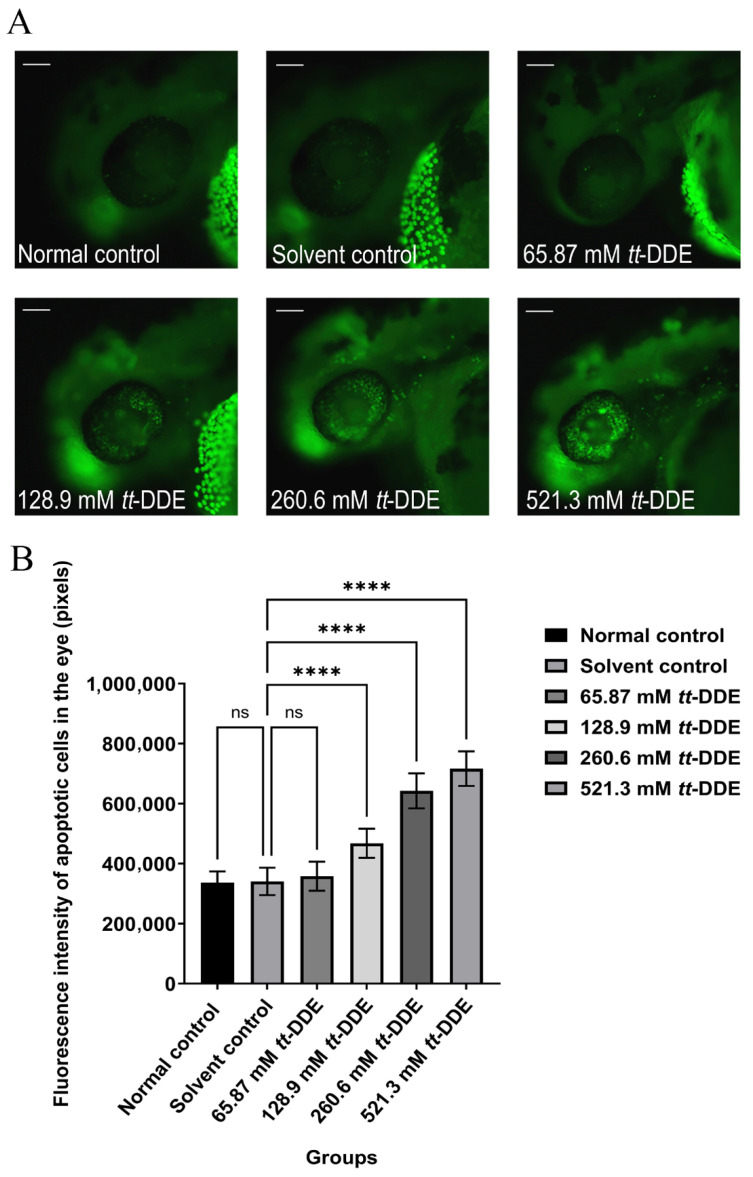
*tt*-DDE induces ocular apoptosis in zebrafish. Wild-type AB zebrafish were ocularly injected with 65.87 mM, 128.9 mM, 260.6 mM, and 521.3 mM of *tt*-DDE; the normal control and the solvent control group were injected with saline and DMSO, respectively. (**A**) Fluorescence micrographs of AO staining in ocular tissue. Increased green fluorescence indicates cells with enhanced membrane permeability and nuclear condensation. Scale bar = 100 μm. (**B**) Quantification of mean fluorescence intensity (in pixels). Data are presented as mean ± SEM, *n* = 10 larvae per group. Statistical comparisons between each tt-DDE-treated group and the solvent control were performed using one-way ANOVA followed by Dunnett’s post hoc test. **** *p* < 0.0001 compared with the solvent control; ns, not significant (*p* > 0.05).

Our results show that treatment with *tt*-DDE induced a significant and dose-dependent increment in AO fluorescence intensity within the zebrafish eye. This effect was visually apparent in Figure 4A, which displayed a *tt*-DDE concentration-dependent increase in green fluorescent apoptotic cells, a finding that was quantitatively confirmed by a corresponding rise in fluorescence intensity (Figure 4B). Although the lowest dose (65.87 mM) resulted in an increase that was not statistically significant compared to the solvent control, the three higher doses (128.9, 260.6, and 521.3 mM) triggered a highly significant escalation in fluorescence intensity (*n* = 10, *p* < 0.001).

### 3.5. Cell Viability

**Figure 5:**
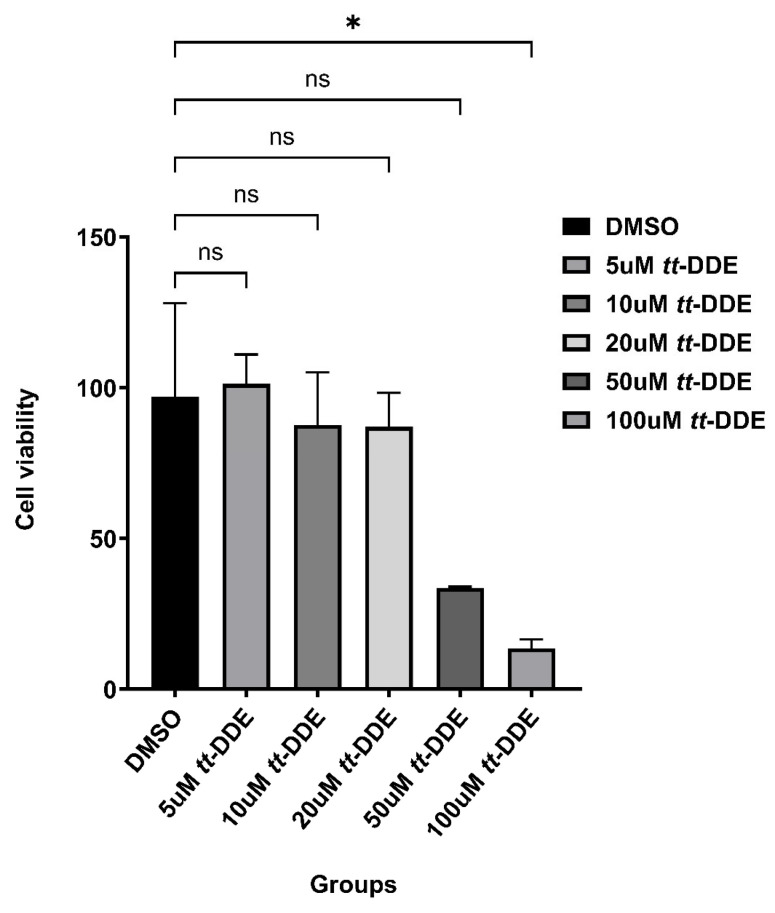
Cytotoxic effects of *tt*-DDE on human retinal endothelial cells (HRECs). HRECs were treated with 0.05% DMSO (solvent control), 5 μM, 10 μM, 20 μM, 50 μM, and 100 μM *tt*-DDE for 24 h. Data are presented as median ± IQR, *n* = 3 independent biological replicates per group. Statistical comparisons between each *tt*-DDE treated group and the solvent control were performed using Kruskal–Wallis non-parametric test followed by Dunn’s post hoc test. * *p* < 0.05 compared with the DMSO group; ns, not significant (*p* > 0.05).

As shown in Figure 5, exposure to *tt*-DDE for 24 h caused a dose-dependent decrease in HREC viability. Compared to the vehicle control, treatment with 5, 10, 20, 50, and 100 μM *tt*-DDE exhibited reductions in cell viability by 3.01%, 11.27%, 6.34%, 73.00%, and 90.25%, respectively. While our statistical analyses indicated no notable disparities between the solvent control group and the lower concentrations (5, 10, 20, 50 μM), significant cytotoxicity was evident at the higher levels of 100 μM (*p* < 0.05).

## 4. Discussion

The present study provides compelling evidence that early-life exposure to *tt*-DDE, a prominent aldehyde in COF, induces severe and dose-dependent impairments in ocular development and angiogenesis in zebrafish larvae. We found that direct exposure to *tt*-DDE resulted in a significant reduction in eye size, inhibition of intraocular vascularization, disruption of retinal histoarchitecture, and a marked increase in acridine orange fluorescence intensity suggestive of enhanced cell death. Furthermore, we confirmed the direct cytotoxic effect of *tt*-DDE on HRECs in vitro. These findings collectively suggest that early-life exposure to *tt*-DDE impairs ocular development in zebrafish larvae through effects involving reduced eye size, inhibited vascular development, increased retinal toxicity, and enhanced cell death, highlighting a significant potential public health concern regarding exposure to cooking oil emissions.

The ocular system has emerged as a sensitive target for environmental pollutants, particularly for the indoor air pollutants [25,26]. As a major *α,β*-unsaturated aldehyde component of COF generated through lipid peroxidation [27], *tt*-DDE poses a significant risk of human exposure. While its toxicity to the cardiovascular, respiratory, and digestive systems is well-documented [8,28], its impact on ocular development has remained largely unexplored. Our study directly addresses this concern by investigating the association between *tt*-DDE and intrinsic ocular developmental toxicity.

Our results demonstrated a pronounced dose-dependent decrease in eye size in zebrafish exposed to *tt*-DDE, indicating its inhibitory effect on ocular development. Furthermore, observations using *fli1:EGFP* transgenic embryos revealed a significant decrease in intraocular vascular area in the *tt*-DDE-treated groups, suggesting a direct toxic effect on angiogenesis, which might be mediated through direct endothelial damage and interference with metabolic pathways. It has been hypothesized that *tt*-DDE may interfere with angiogenesis by affecting vascular endothelial growth factor or regulating related signaling pathways [29,30,31]. Additionally, *tt*-DDE may indirectly exacerbate microangiopathy by dysregulating the insulin signaling pathway, suggesting a complex interplay between metabolic and vascular homeostasis [32]. A recent study using aldh9a1b knockout zebrafish reported that *tt*-DDE accumulation induced retinal hypervascularization and that this phenotype could be rescued by insulin sensitizers such as metformin and rosiglitazone [33]. These findings support the hypothesis that insulin resistance may serve as a link between *tt*-DDE exposure and ocular vascular defects. The relationship between *tt*-DDE exposure and endothelial dysfunction has been documented in prior studies. The compound has been shown to induce endothelial dysfunction by disrupting cell–cell junctions, increasing vascular permeability, and promoting a pro-inflammatory milieu [5,34,35]. These documented effects provide a plausible biological basis for the reduced intraocular vascularization observed in our zebrafish model, although the specific molecular pathways involved in the ocular context remain to be elucidated through targeted experimental approaches.

Apoptosis, a form of programmed cell death, plays a pivotal role in ocular development and disease pathogenesis. Earlier research established that COF can induce apoptosis in cells through the MAPK/NF-κB/STAT1 pathway [36]. In this study, AO staining revealed that fluorescence intensity in zebrafish eyes increased in a dose-dependent manner after exposure to *tt*-DDE. Elevated AO fluorescence is associated with changes in membrane permeability and nuclear condensation, which are features of cell death [24]. Our finding is consistent with previous reports showing that *tt*-DDE triggers mitochondria-dependent apoptosis of human corneal epithelial cells [9]. The potential mechanisms may involve activation of endoplasmic reticulum stress, generation of reactive oxygen species, and depletion of mitochondrial membrane potential [9]. These proposed mechanisms are supported by existing literature on *α*,*β*-unsaturated aldehydes, which have been shown to cause vascular dysfunction and induce apoptosis through oxidative stress and mitochondrial damage [34]. As a tissue with high oxygen consumption, the retina has a significantly higher mitochondrial density than other ocular structures [37], potentially making it more susceptible to oxidative damage by *tt*-DDE. Additionally, histological sections revealed chromatin margination and nuclear pyknosis, further supporting activation of the endogenous apoptotic pathway [38]. These results indicate that apoptosis may represent one of the significant mechanisms underlying *tt*-DDE-induced ocular toxicity, though this interpretation should be regarded as preliminary pending further verification.

After exposure to *tt*-DDE, the proliferative capacity of HRECs was significantly reduced, manifested as a dose-dependent decrease in cell viability. This finding aligns with our zebrafish results of reduced ocular vascularization and retinal structural disruption. Previous studies have shown that *tt*-DDE, as a toxic product of lipid peroxidation, can induce cytotoxicity and lethal effects in various models, including human fibroblasts, endothelial cells, and erythroleukemia cells [39,40,41]. For example, in human erythroleukemia cells, *tt*-DDE led to a marked increase in cellular glutathione (GSH) levels at early time points followed by a sharp decline, accompanied by the appearance of low molecular weight DNA fragments, suggesting that GSH depletion and DNA damage contribute to its cytotoxicity [41]. Previous research reported that *tt*-DDE exacerbates apoptosis by upregulating the expression of caspase-3/9 and activating the JNK signaling pathway, supporting that *tt*-DDE-induced apoptosis might be the primary cause of the decreased cell proliferation capacity [10]. Similarly, *tt*-DDE significantly inhibited the viability of HUVECs by impairing mitochondrial function and autophagic flux [5]. The convergence of evidence from multiple cell types supports the notion that *tt*-DDE exerts broad cytotoxic effects, and our HRECs data extend these observations to the retinal microvasculature. The consistency between the in vivo zebrafish phenotypes and the in vitro HRECs responses strengthens the biological plausibility of our findings, while also underscoring the need for mechanistic studies to identify the specific molecular targets of *tt*-DDE in retinal endothelial cells.

While our study offers preliminary evidence regarding the impact of *tt*-DDE on ocular development, limitations must be acknowledged. First, although direct ocular injection of *tt*-DDE effectively demonstrates dose-dependent ocular toxicity, this route of exposure does not fully mimic the aerosolized nature of cooking oil fumes encountered in human occupational or domestic settings. Caution is warranted when extrapolating our findings directly to human risk assessment. Second, this study focused exclusively on the acute exposure effects, whereas chronic toxicity also holds significant implications. Prior research has shown that prolonged low-dose *tt*-DDE can lead to hyperproliferation of human bronchial epithelial cells [42]. Moreover, our study did not delve deeply into the mechanistic underpinnings of the observed toxic effects. Finally, the reliance on a single in vitro model may not adequately capture the complex biological responses that occur in vivo. Therefore, future studies should systematically evaluate the effects of chronic exposure to subthreshold concentrations on eye development in zebrafish models and further explore the chronic cytotoxic effects and mechanisms.

## 5. Conclusions

In summary, to our knowledge, this is the first study to demonstrate the ocular toxicity of the cooking oil fume component *tt*-DDE in a zebrafish model and establish an in vitro model using retinal endothelial cells to study its damaging effects. While intraocular injection does not directly replicate human inhalation exposure, the results confirm the intrinsic retinotoxic potential of *tt*-DDE. Our findings consistently revealed a dose-dependent impairment of eye development linked to *tt*-DDE, including a reduction in eye size, inhibition of intraocular angiogenesis, and histopathological alterations characterized by increased apoptotic-like changes and disrupted retinal histoarchitecture. The corroborating in vitro evidence of cytotoxicity in HRECs underscores the potential translational relevance of these findings and suggests a direct toxic effect of *tt*-DDE on vascular endothelial cells. Given the structural and functional similarities between zebrafish and human ocular systems, existing data indicate that this compound possesses intrinsic eye toxicity under direct exposure conditions. Further investigation is needed to assess whether cooking-related airborne pollutants pose ocular health risks to humans under environmentally relevant exposure scenarios.

## Data Availability

The original contributions presented in this study are included in the article/Supplementary Materials. Further inquiries can be directed to the corresponding authors.

## Supplementary Materials

The following supporting information can be downloaded at: https://www.mdpi.com/article/10.3390/toxics14050388/s1, Table S1. Mortality and phenotypic outcomes in zebrafish after ocular administration of different doses of *tt*-DDE and control vehicle. Figure S1. Dose-mortality curve of *tt*-DDE in zebrafish following intraocular injection.
