# Supplementary material for: Early-Life Exposure to the Cooking Oil Fume Component trans,trans-2,4-Decadienal Impairs Ocular Development and Angiogenesis in Zebrafish (Danio rerio) Larvae
Source: Toxics. 2026 Apr 30;14(5):388. doi: 10.3390/toxics14050388 (PMC13211680; doi:10.3390/toxics14050388)
Supplement: Supplementary file 1 [file toxics-14-00388-s001.zip › toxics-4250180-supplementary.pdf]

Supporting Information

Early-Life Exposure to the Cooking Oil Fume Component *trans,trans*-2,4-Decadienal Impairs Ocular Development and Angiogenesis in Zebrafish (*Danio rerio*) Larvae

Xiaoli Wu [1,2,†], Xinyue Zhang [1,2,†], Zengliang Ruan [1,2,3*]

[1] Key Laboratory of Environmental Medicine and Engineering of Ministry of Education, School of Public Health, Southeast University, Nanjing, China

[2] Department of Epidemiology and Health Statistics, School of Public Health, Southeast University, Nanjing, China

[3] Department of Medical Epidemiology and Biostatistics, Karolinska Institutet, Stockholm, Sweden

*Correspondence and requests for materials should be addressed to Zengliang Ruan (E-mail:rzl@seu.edu.cn). Tel.: +86-25-83272561.

Table S1. Mortality and phenotypic outcomes in zebrafish after ocular administration of different doses of *tt*-DDE and control vehicle.

Group	Dosage (M)	Deaths (tail)	Mortality (%)	Phenotype
**Control**	-	0	0	No abnormality
**Vehicle**	-	0	0	No abnormality
***tt*-DDE**	0.3580	0	0	Similar to Vehicle
	0.7160	0	0	Similar to Vehicle
	1.4321	10	33	-
	2.8641	20	67	-
	5.7282	30	100	-

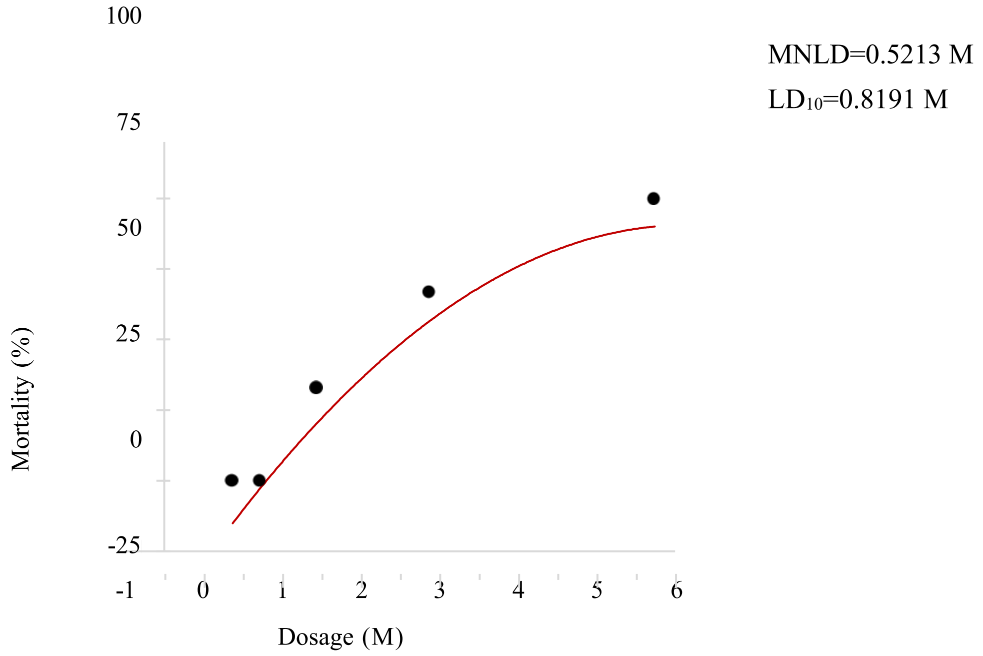

Figure S1. Dose-mortality curve of *tt*-DDE in zebrafish following intraocular injection.
